# The podocyte: glomerular sentinel at the crossroads of innate and adaptive immunity

**DOI:** 10.3389/fimmu.2023.1201619

**Published:** 2023-07-26

**Authors:** George W. Burke, Alla Mitrofanova, Antonio Fontanella, Gaetano Ciancio, David Roth, Phil Ruiz, Carolyn Abitbol, Jayanthi Chandar, Sandra Merscher, Alessia Fornoni

**Affiliations:** ^1^ Division of Kidney−Pancreas Transplantation, Department of Surgery, Miami Transplant Institute, University of Miami Miller School of Medicine, Miami, FL, United States; ^2^ Research, Katz Family Division of Nephrology and Hypertension, Department of Medicine, University of Miami Miller School of Medicine, Miami, FL, United States; ^3^ Katz Family Division of Nephrology and Hypertension, Department of Medicine, and the Miami Transplant Institute, University of Miami Miller School of Medicine, Miami, FL, United States; ^4^ Transplant Pathology, Department of Surgery, Miami Transplant Institute, University of Miami Miller School of Medicine, Miami, FL, United States; ^5^ Division of Pediatric Nephrology, Department of Pediatrics, University of Miami Miller School of Medicine, Miami, FL, United States; ^6^ Division of Pediatric Kidney Transplantation, Department of Pediatrics, Miami Transplant Institute, University of Miami Miller School of Medicine, Miami, FL, United States; ^7^ Katz Family Division of Nephrology and Hypertension, Department of Medicine, University of Miami Miller School of Medicine, Miami, FL, United States

**Keywords:** podocyte, kidney, adaptive immunity, innate immunity, immune pathway

## Abstract

Focal segmental glomerulosclerosis (FSGS) is a common glomerular disorder that manifests clinically with the nephrotic syndrome and has a propensity to recur following kidney transplantation. The pathophysiology and therapies available to treat FSGS currently remain elusive. Since the podocyte appears to be the target of apparent circulating factor(s) that lead to recurrence of proteinuria following kidney transplantation, this article is focused on the podocyte. In the context of kidney transplantation, the performance of pre- and post-reperfusion biopsies, and the establishment of *in vitro* podocyte liquid biopsies/assays allow for the development of clinically relevant studies of podocyte biology. This has given insight into new pathways, involving novel targets in innate and adaptive immunity, such as SMPDL3b, cGAS-STING, and B7-1. Elegant experimental studies suggest that the successful clinical use of rituximab and abatacept, two immunomodulating agents, in our case series, may be due to direct effects on the podocyte, in addition to, or perhaps distinct from their immunosuppressive functions. Thus, tissue biomarker-directed therapy may provide a rational approach to validate the mechanism of disease and allow for the development of new therapeutics for FSGS. This report highlights recent progress in the field and emphasizes the importance of kidney transplantation and recurrent FSGS (rFSGS) as a platform for the study of primary FSGS.

## 1 Introduction

Legend has it that Blues guitarist extraordinaire Robert Johnson sold his soul to the devil in return for being taught how to play the guitar. The deal was consummated at the crossroads of Highway 49 (known as the Blues Highway) and Route 61 in Clarksdale, Mississippi in 1927. Robert Johnson went on to become the father of American blues music, The King of the Delta Blues, spawning a generation and genre of African-American Blues musicians. It remains to be determined whether the podocyte in being positioned also at the crossroads of innate and adaptive immunity, was by divine intervention, or perhaps a deal made with the devil, similar to that of Robert Johnson. This is of more than passing interest, since it may well relate to podocyte longevity and lifetime/survival. You see, Robert Johnson died at the age of 27, starting a sad legacy of rock musicians who did not make it past their 27th birthday (see **Supplemental Appendix**). In a similar fashion, the podocyte may live out its functional existence in a normal fashion, or if it is involved in a disease process, for example focal segmental glomerulosclerosis (FSGS), may die young, the equivalent of an unfortunate 27 year-old rock musician. Please indulge this metaphysical conceit (with appreciation to John Donne), placing glomerular podocytes involved in FSGS in a position similar to doomed rock musicians. It is particularly fitting that it was Robert Johnson who wrote the song “Crossroads” (originally “Cross Road Blues”), emphasizing for us the intersection of two key immune pathways. Our mission is to determine how best to protect, preserve, and/or restore the life of our kidney rock star, the podocyte. 

Focal segmental glomerulosclerosis (FSGS) is a common glomerular disorder that manifests clinically with nephrotic syndrome and > 80% foot process effacement on electron microscopy (1). FSGS accounts for about 20% of cases of nephrotic syndrome in children and 40% in adults, and is the most common glomerular disorder leading to end stage kidney disease (ESKD) in the United States with a prevalence of 4% (2). Progression to ESKD occurs in 40-60% of patients within 10-20 years from diagnosis, making this the most common primary glomerular disease leading to dialysis in the United States (3). Despite many clinical trials, no effective treatment has been identified (4). Following transplantation, recurrence of FSGS occurs in 20-50% of adults (2, 3, 5) and up to 80% in high-risk pediatric patients (6). Risk factors for recurrent FSGS include younger age (children less than 6 at onset), non-black race, rapid progression to ESKD (less than 3 years from diagnosis), severe proteinuria immediately prior to transplantation, and the loss of a previous allograft(s) to recurrence (2, 3). Recurrence of FSGS increases the risk of renal dysfunction and early graft loss (7, 8).

## 2 Pathophysiology of FSGS recurrence

### 2.1 Circulating factor(s)

Recurrence of FSGS can develop rapidly after transplantation, sometimes within minutes to hours (9), suggesting the presence of a circulating factor(s) that could mediate an early injury to the glomerulus. Further evidence includes the following: 1) plasmapheresis can induce remission of proteinuria (5), and 2) serum from patients with FSGS induces proteinuria in rats, and alters albumin permeability in isolated glomeruli (2, 10). Moreover, in two clinical reports, living donor kidney transplant recipients who experienced severe recurrent FSGS, underwent transplant nephrectomy with re-transplantation of the affected kidney into a non-FSGS recipient (11, 12) resulting in rescue of the donor kidney function with resolution of proteinuria, providing further evidence for a circulating factor(s). Several review articles provide details supporting the existence of circulating factors that are beyond the scope of this article (13, 14).

### 2.2 Immunology of FSGS

Despite the lack of evidence of immune infiltrates in human FSGS biopsy samples (2, 6), an immune component has been thought to contribute to FSGS (15, 16). For example, the identification of IgM and C3 in glomerular deposits which was associated with both unfavorable response to therapy and worse renal outcomes (17) supports this concept. T-cell involvement has been described in the Buffalo/Mna rat model of FSGS recurrence after renal transplantation (18), and in human recurrent FSGS needle aspirate samples (19). However, neither B cell nor T cell infiltrates are consistently found in FSGS (20). Nonetheless, immunosuppression, which was associated with reduced proteinuria in the Buffalo/Mna rat model (18), has been the main therapy in humans after plasmapheresis or plasma absorption. The most studied immune-directed therapies have included corticosteroids, inhibitors of T lymphocyte calcineurin (cyclosporine A and tacrolimus), alkylating agents (cyclophosphamide and chlorambucil), and mycophenolate mofetil (5, 6). A number of case reports and non-controlled series have reported benefit from rituximab (8, 21, 22) but this has not been universally successful (23). Similarly, it remains unclear if the benefit of these immunosuppressive agents is linked to immunomodulatory functions or to direct podocyte effects.

### 2.3 At the crossroads of innate and adaptive immunity

#### 2.3.1 Podocyte innate immunity

The glomerular capillary wall consists of three layers: 1) the fenestrated endothelial cell, 2) the glomerular basement membrane (GBM), and 3) the podocyte, the last barrier to prevent loss of protein into Bowman’s space. Thus, the podocyte is anatomically located in a position to be a sentinel for the kidney’s innate immune system. The mammalian innate immune system was originally intended to protect its host from outside pathogens, for example viruses, bacteria and fungi – pathogen associated molecular patterns (PAMP’s) (24) and in the process distinguish self from non-self. The theory of immunity has since evolved to the recognition of danger signals proposed by Polly Matzinger (25) and more recently to the discontinuity theory (26, 27): that immune activation can be induced by changes (any) in the environment or the cell rather than by detection of specific, defined molecules (24). Ultimately, the innate immune response requires a balance between the appropriate degree of aggression in the face of a serious pathogenic threat, but also the ability to limit local tissue damage with a controlled response.

**Figure 1:**
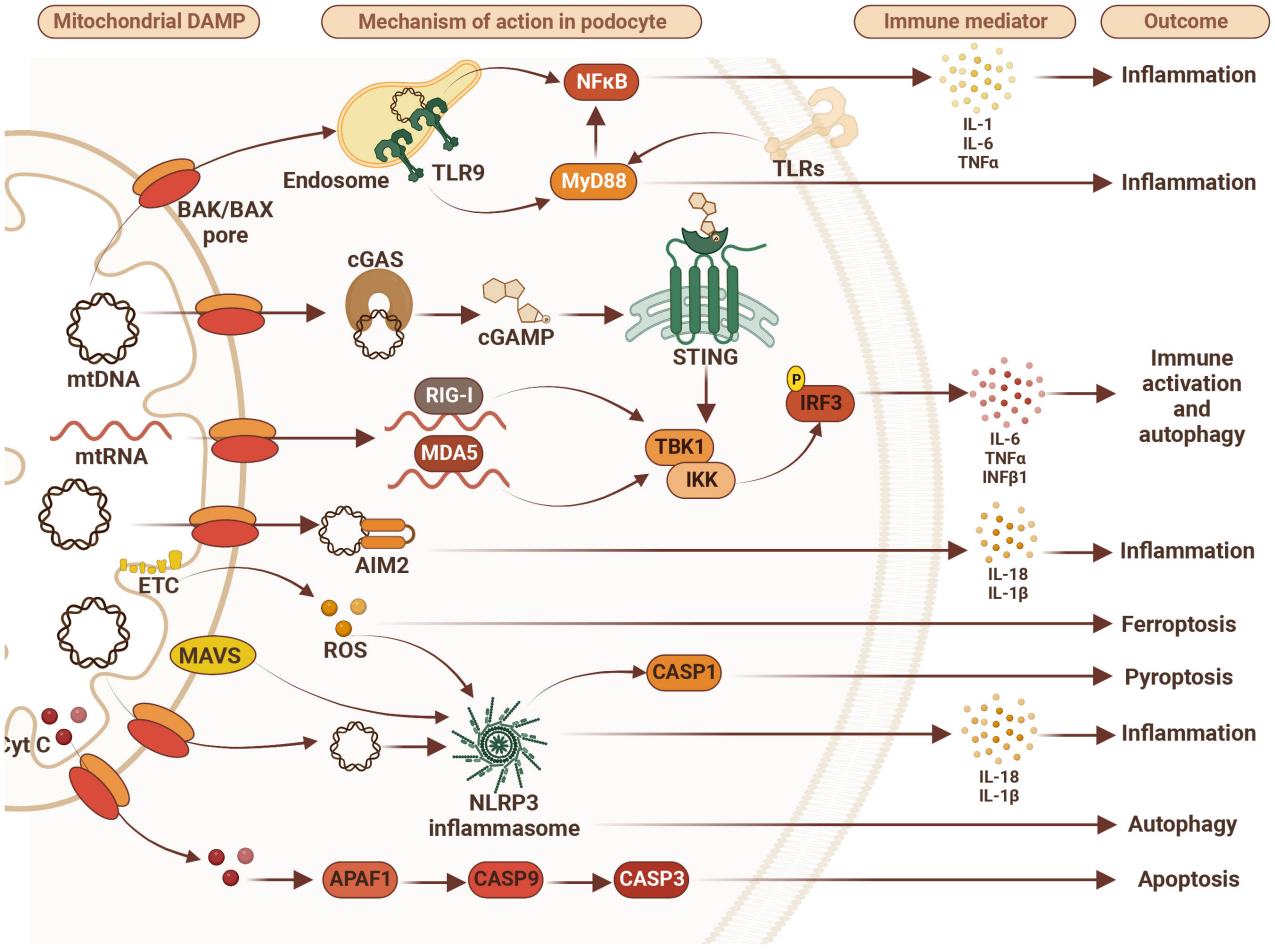
Mitochondrial damage-associated molecular patterns (DAMPs) disturb podocyte innate immunity pathways. Mitochondrial DAMPS such as mitochondrial DNA (mtDNA), mitochondrial RNA (mtRNA), cytochrome C (Cyt C), and reactive oxygen species (ROS) contribute to disturbance in podocyte homeostasis via activation of various DNA/RNA-sensing and inflammasome pathways leading to inflammation, immune system activation and different types of cell death including apoptosis, ferroptosis and pyroptosis. Therefore, targeting immune markers such as toll-like receptors (TLRs), tumor necrosis factor alpha (TNFα), NLR family pyrin domain containing 3 (NLRP3), retinoic acid-inducible gene I (RIG-I), melanoma differentiation-associated protein 5 (MDA5), mitochondrial antiviral-signaling protein (MAVS), absent in melanoma 2 (AIM2), stimulator of interferon gene (STING), and/or myeloid differentiation primary response 88 (MyD88) may represent future therapeutic approaches to treat FSGS. AIM2 – absent in melanoma 2; APAF1, apoptotic pepdidase activating factor 1; BAK, Bcl2 homologous antagonist/killer; BAX, Bcl2-associated X protein; CASP1, caspase 1; CASP3, caspase 3; CASP9, caspase 9; cGAS, cyclic GMP-AMP synthase; cGAMP, 2’3’-cyclic GMP-AMP; Cyt C, cytochrome c; ETC, electron transport chain; IL-1β, interleukin 1β; IL6, interleukin 6; IL-18, interleukin 18; IKK, IkB kinase; IRF3, interferon regulatory factor 3; MDA5, melanoma differentiation-associated protein 5; NF-kB, activation of nuclear factor κB; NLRP3, NLR family pyrin domain containing 3; RIG-I, retinoic acid-inducible gene I; ROS, reactive oxygen species; STING, stimulator of interferon gene; TBK1, TANK binding kinase 1; TLR9, toll-like receptor 9; TNFα, tumor necrosis factor alpha.

Podocytes have been shown to express Toll-like receptors (TLRs), which recognize PAMPs, and certain endogenous signals, for example cellular breakdown products (Damage-Associated Molecular Patterns– DAMPs), e.g. reactive oxygen species, mitochondrial or, endoplasmic reticulum stress, DNA, RNA, nucleic acid fragments, etc. (28). TLRs which bind to DAMP’s/PAMP’s are part of the cellular (host) innate immune defense system, which, once activated, induce secretion of specific cytokines and chemokines that prompt the recruitment of other components of the innate immune response (29, 30). TLR stimulation activates nuclear factor Kappa B (NF Kappa B) in podocytes, and key components of TLR signaling including MyD88, IRAK, TRAF 6, etc. (**Figure 1**) (31). It has been proposed that the podocyte’s role in innate immunity may predispose it to injury, depending on chronicity (32).

This is supported by the demonstration that TLR stimulation leads to podocyte apoptosis, and that blocking this pathway may prevent the progression of fibrosis and disease (33). In addition to TLRs there are a number of other important Pattern Recognition Receptors (PRRs) that are expressed by podocytes. These include the major components of the inflammasome, Nod-like receptors (NLRP3), ASC and caspase 1 (34, 35). The inflammasome is an intracellular protein complex involved in innate immune function including autophagy, apoptosis, fibrosis and secretion of pro-inflammatory cytokines including IL1-Beta and IL18 (28–30, 32). Podocytes also express retinoic acid-inducible gene 1 (RIG-1) that belongs to the family of RIG-I-like helicases that recognize viral RNA (32). Other family members include MDA5 and upon activation by recognition of viral RNA by cytoplasmic sensors, signal to the mitochondrial adaptor MAVS inducing an Interferon-regulatory factor (IRF)3, and IRF7-mediated type 1 IFN response (**Figure 1**) (35, 36).

Recently, the cGAS-STING axis has been identified as playing an important role in podocyte innate.

Immunity (37). cGAS, a PRR, is considered to be a key sensor of cytosolic DNA leading to catalyzation of second messenger 2’3’-cGAMP, which binds to the endoplasmic reticulum membrane adaptor STING (encoded by TMEM173) leading to activation of TANK-binding kinase 1 (TBK1), which in turn activates nuclear interferon regulatory factor 3 (IRF3) and leads to the production of type I interferons (**Figure 1**) (38, 39). The development of the cGAS-STING cytoplasmic defense system provides recognition of foreign nucleic acids, including pathogens/PAMPs (39), and DAMPs, as well as self-mitochondrial DNA leakage (40), cellular stress (24), and senescent break down products (41). This remarkably complex and efficient system based on the spatial coordination of DNA induced-cGAS-STING activity (38) allows for antiviral immunity and balanced innate immune responses toward self-DNA. While the crucial roles of the cGAS-STING pathway in immune cells and the immune defense have been extensively investigated (24, 38, 39), its function in non-immune cells, such as podocytes, remains largely unknown. It is not known if STING contributes to the development and/or progression of glomerular diseases, however, our group has been investigating this, and our work is summarized in this article (37).

### 2.4 Adaptive immune response

Beyond its role in innate immunity, the podocyte is capable of contributing to the adaptive immune response (16). The podocyte has been shown to possess properties of non-hematopoietic professional antigen-presenting cells (APCs**)** (42). Expression of both class I MHC antigens and, under certain inflammatory conditions, MHC class II antigens, contributing to the activation of CD8 and CD4 positive T cells, respectively, has been demonstrated on the podocyte. Moreover, podocytes express B7-1 (also known as CD80), a co-stimulatory molecule for T cells which is inducible under conditions of stress and was first reported in the context of the nephrotic syndrome (43). B7-1/CD80 is part of the B cell and APC repertoire and provides the second signal to T cells that allows amplification of the response to peptide antigens presented in the context of the appropriate MHC class I/II background (signal 1). B7-1 binds to CD28 on the T cell membrane, resulting in a positive co-stimulatory signal (signal 2). Interestingly, B7-2 (CD86), a co-stimulatory molecule presents on B cells and APCs that is also capable of binding to CD28 on T cells, is not present on podocytes (43).

The podocyte is in a position to integrate and act upon the range of immune signals, from T/B cell secreted cytokines to DAMPs/PAMPs. When podocytes are activated, through TLR’s, cGAS-STING, or B7-1, etc., the change, mediated through the actin cytoskeleton, can affect the slit diaphragm proteins (44), allowing the passage of protein (proteinuria) or potentially danger signals, e.g. pathogens, into Bowman’s space, and hence into the urine and excreted (29, 30). This potential for the excretion of danger/pathogen imbues the kidney, by virtue of the podocyte, with the capacity for immune defense mediated through a combination of innate and adaptive immune mechanisms (16). Importantly, clearance of danger into the urine avoids the activation of inflammatory mediators/cytokines that could result in unnecessary local tissue damage (45).

## 3 Treatment strategies: immunomodulation vs. direct podocyte effect of rituximab for recurrent FSGS

Rituximab is a monoclonal antibody directed against CD20 expressed on B lymphocytes and has been used for the treatment of non-Hodgkin’s lymphoma, chronic lymphocytic leukemia and rheumatoid arthritis (46). Rituximab has been used off-label for the treatment of several kidney conditions, including acute allograft rejection and steroid resistant nephrotic syndrome (46). However, the pathogenesis of FSGS has not been demonstrated to be B cell or antibody mediated, suggesting the possibility of a B lymphocyte – independent mechanism of action. To assess the possible role of rituximab in recurrent FSGS, our center began utilizing a single dose of rituximab peri-operatively at the time of kidney transplantation in January 2004. In this study 27 consecutive patients received rituximab and were compared to a historical control group of 14 patients. This group of kidney transplant recipients was comprised predominately of children at high risk for recurrence (47). Nephrotic range proteinuria within the first month post- transplant was reduced from 64% to 26% (p=0.023), and similarly the need for plasmapheresis fell from 71% to 30% (p=0.019).

### 3.1 Peri-operative use of rituximab: beyond B cell immunity

In an attempt to better understand the mechanism of rituximab in this clinical setting, we pursued two lines of research: one involving pre- and post-reperfusion kidney transplant biopsies, and the other based on the effect of patient serum on podocytes in an *in vitro* assay. We initially expected to identify CD20 on the podocyte membrane, however, we demonstrated direct binding of rituximab to podocytes in the absence of CD20 (47). Screening of a phage display peptide library revealed possible cross reactivity of rituximab with sphingomyelin phosphodiesterase acid-like 3b (SMPDL3b) protein (48). Exposure of lymphoma cells to rituximab regulates the action of acid sphingomyelinase in lipid raft microdomains (49) essential for the organization of signaling molecules in highly specialized cells like podocytes. Based on these findings, we hypothesized that rituximab might have an impact on the filtration barrier in recurrent FSGS via the stabilization of sphingolipids with downstream effect on actin cytoskeleton remodeling. We postulated that rituximab could act as a direct modulator of podocyte function similar to what has been reported for cyclosporine, that was found to modulate podocyte function by interacting with the podocyte cytoskeleton, independent of its immunosuppressive activity (50).

As part of the protocol for kidney transplantation, a preimplantation biopsy of the allograft was obtained followed by a second biopsy 30 to 60 minutes post-reperfusion, after the completion of the uretero-vesical anastomosis, prior to closure of the incision. We demonstrated membrane and lipid raft specific SMPDL3b expression on podocytes from normal donor kidney biopsies (pre-reperfusion). Interestingly, the number of SMPDL3b-positive podocytes in post-reperfusion biopsies was reduced in patients who later experienced recurrent proteinuria (47). Serum collected in the pretransplant setting from patients who developed recurrent proteinuria resulted in down regulation of SMPDL3b protein expression and acid sphingomyelinase activity in cultured immortalized human podocytes. This was blocked by pretreatment of the podocytes with rituximab. Similarly, pretransplant serum of patients with recurrent disease caused a marked disruption of the actin cytoskeleton which could be prevented by pretreatment with rituximab, or overexpression of SMPDL3b on the podocytes in culture. The protective effect of rituximab was blocked by the addition of siRNA to SMPDL3b (47). Thus, down regulation of SMPDL3b after exposure to patient’s serum rendered podocytes more susceptible to actin remodeling, likely caused by a circulating/permeability factor(s). Rituximab appeared to prevent podocyte actin remodeling through stabilization of SMPDL3b (51). In addition, we reported that SMPDL3b may be an important modulator of podocyte function by influencing suPAR-mediated podocyte injury, changing it from a migratory to an apoptotic phenotype (52).

In summary, we have demonstrated that rituximab treatment of high-risk FSGS kidney transplant recipients is associated with a lower incidence of post-transplant proteinuria and could directly protect podocytes in an SMPDL3b-dependent manner (47). The demonstration of SMPDL3b-mediated protection from proteinuria using rituximab in a kidney transplant xenograft (53) and adriamycin-induced nephropathy model (54) further support this. SMPDL3b has also been shown to interact with TLRs and play an important role in down-regulating innate immunity (55).

### 3.2 Changes identifiable on post-reperfusion biopsies

In those patients who experienced recurrent proteinuria, we identified podocyte foot process effacement by electron microscopy in the post-reperfusion biopsies. Furthermore, we were able to show that the presence of foot process effacement correlated with the degree of proteinuria (9). However, resolution of foot process effacement in the context of recurrent FSGS was not always associated with resolution of proteinuria (56, 57). This suggests that proteinuria related to disruption of the slit diaphragm proteins may be a more subtle injury than that recognized by foot process effacement (9) on electron microscopy. The changes identifiable in the post-reperfusion biopsy emphasize the likelihood of the existence of circulating factor(s) in the kidney recipients with FSGS (9).

### 3.3 Abatacept rescue in the context of podocyte B7-1 staining

The use of rituximab peri-operatively in high-risk kidney transplant recipients with FSGS resulted in significant reduction in the development of recurrent proteinuria (47). However, 26% of the patients developed nephrotic-range proteinuria despite receiving a single dose of rituximab. This prompted us to further explore the possibility of other podocyte-directed treatment. Based on the demonstration of B7-1/CD80 expression in podocytes that was initially reported in 2004 (43) in the context of the nephrotic syndrome, we stained our pre- and post-reperfusion biopsies in KT recipients with recurrent proteinuria for B7-1. We subsequently identified B7-1 on the post-, but not the pre-reperfusion biopsies. Abatacept (CTLA-4-Ig) is an inhibitor of the T cell costimulatory molecule B7-1 (CD80) and is used to treat rheumatoid arthritis (58). Importantly, abatacept possesses a greater affinity for B7-1 than B7-2 (59). Since podocytes selectively express B7-1, and not B7-2 (43) as described above, we hypothesized that abatacept might be particularly effective in our unique clinical context.

Furthermore, we performed detailed mechanistic *in vitro* studies of podocytes, similar to our efforts with rituximab (47). Podocyte migration *in vitro* is known to correlate with function *in vivo*. We demonstrated that the addition of LPS to podocytes in culture induced B7-1 protein expression and affected migration. This was blocked by abatacept. In addition, B7-1 gene silencing or expression of the truncated construct also suppressed podocyte migration. B7-1 positive podocytes have a reduced capacity to attach to the surrounding matrix through beta 1 integrin. In cell culture studies, B7-1 positive podocytes changed their morphological characteristics and their function, leading to detachment of podocyte foot processes from the GBM. Abatacept appears to act by blocking the B7-1-mediated loss of podocyte Beta 1 integrin activation. Mechanistically, B7-1 promotes disease-associated podocyte migration through inactivation of beta 1 integrin, and this is reversed by abatacept (60).

Four kidney transplant recipients who were at high risk for recurrence (two patients with retransplants; two pediatric patients with rapid progression to ESKD), received a single dose of rituximab peri-operatively (47), and experienced post-transplant nephrotic range proteinuria. The first patient was treated with abatacept and plasmapheresis during the first week following transplantation, and experienced resolution of nephrotic range proteinuria. Treatment with abatacept (60) 10 mg/kg IV, resulted in the partial or complete remission of proteinuria in the three other kidney transplant recipients subsequently. In the two patients who underwent pre- and post-reperfusion biopsies, B7-1 staining was shown on the post-, not the pre-reperfusion biopsy, and B7-1 was found to colocalize with synaptopodin. Similar resolution of proteinuria after abatacept therapy was demonstrated in a non-transplant patient with native FSGS resistant to therapy who was shown to have B7-1 positive staining on biopsy (60). Of the four kidney transplant recipients, two received a single dose of abatacept, while the other two received two doses. All four recipients received their first dose of abatacept within one week of transplantation. These patients received induction antibody therapy with Thymoglobulin and monoclonal anti-CD25 (IL2R), and maintenance immunosuppression with tacrolimus, mycophenolate mofetil and steroids, as well as plasmapheresis. With follow-up from ten to forty-eight months, the creatinine remained stable, urine protein/creatinine ratio was < 0.1 for two patients, and < 0.5 in the other two patients (60).

### 3.4 Updated experience with B7-1/abatacept for rFSGS

Since the publication of this experience, a number of articles described difficulty in staining kidney transplant biopsies for B7-1 (61–65) and lack of efficacy of either abatacept or the second-generation anti-costimulatory agent, belatacept (63, 66–69). Over the last decade we have followed 12 patients who experienced recurrent FSGS following KT despite receiving a peri-operative dose of rituximab (70) and found the following:

1) Nine/ten patients with recurrent proteinuria after KT responded to abatacept with improvement or resolution of proteinuria. Of these, two patients did not undergo a KT biopsy. Eight KT recipients were found to be B7-1 positive, one of whom was receiving belatacept at the time of biopsy. This last patient did not respond to abatacept, possibly due to belatacept interference of B7-1 (70).
2) Two patients who remained B7-1 negative on three kidney transplant biopsies during the year following kidney transplantation did not respond to abatacept and lost KT function.
3) None of our patients and no patients from the literature responded favorably to belatacept, regardless of the B7-1 staining (65–69).
4) The resolution of recurrent proteinuria for the second time in two patients who only received abatacept [cases 7 and 8 (70)] adds further strength to support the role for abatacept, since this occurred without plasmapheresis, and in the context of therapeutic calcineurin inhibitor levels (70).

The long-term goal of our program, to optimize treatment of patients with FSGS at risk for recurrent proteinuria after kidney transplantation, has been focused on stratifying patients based on tissue-demonstrated biomarkers. The finding that rituximab binds to SMPDL3b, which is present on healthy podocytes, but is reduced in the context of proteinuria (47) was the first key element. Our clinical experience with rituximab was encouraging, and, to further emphasize the importance of preventing the recurrence of nephrotic range proteinuria, it has been reported that, while a response to rituximab results in 100% five-year graft survival, a lack of response leads to only 36.5% five-year graft survival (8). This concern contributed to our search for a rescue therapy for those kidney transplant recipients who experienced recurrent proteinuria despite receiving a dose of rituximab. In a similar effort to implement biomarker-specific therapy, abatacept, which binds B7-1/CD80, was used in those kidney transplant recipients whose proteinuria recurred despite receiving a peri-operative dose of rituximab. This was done in association with kidney transplant biopsies (when available) to identify podocyte B7-1/CD80 expression (60). In those instances where a kidney transplant biopsy was performed, B7-1 staining was identified on the post-reperfusion, not the pre-reperfusion KT biopsy and treatment with abatacept resulted in improvement/resolution of the proteinuria. This included one example of abatacept treatment-related resolution of proteinuria where B7-1 was demonstrated on a kidney transplant biopsy (70) in which the native kidney disease was not FSGS (series case #3). This suggests that the presence of B7-1 on biopsy in the context of nephrotic range proteinuria may be sufficient to justify treatment with abatacept, regardless of the original disease. Nonetheless, it will be important to continue to study this patient population to confirm the role of both B7-1 expression on podocytes and clinical response to abatacept in those cases of B7-1 positivity.

## 4 Back to the crossroads: the intersection of innate and adaptive immunity

### 4.1 Role of SMPDL3b in podocyte cell membrane function

Our experience treating patients with recurrent proteinuria after receiving a kidney transplant for FSGS has led to further study searching for evidence implicating podocyte involvement in the innate immune response. Human podocytes express TLR 1-6 and 9 mRNA [with minimal expression of TLR 7,8,10 (71, 72)], as well as the membrane sphingolipid SMPDL3b (47, 71). In dendritic cells TLR stimulation, for example TLR 4 with LPS, but also with different ligands including CPG, imiquimod, and the pro-inflammatory cytokine IFN-gamma, results in up-regulation of SMPDL3b mRNA and protein levels (55, 73). SMPDL3b plays a negative role in the regulation of TLR activity *in vivo* associated with reduction in IL-6, TNF-alpha, and IL-12p40 (55). SMPDL3b depletion results in profound changes in the cellular lipid composition and membrane fluidity *in vitro*. SMPDL3b-deficient mice show enhanced responsiveness in TLR-dependent peritonitis. Interestingly, the introduction of specific ceramides could restore regulatory control of TLR stimulation in the context of SMPDL3b depletion (54). These features demonstrate the important role of SMPDL3b in cell membrane lipid composition and flexibility linking this with innate immune signaling (55).

There are suggestions that sphingolipids may also be involved in the development of proteinuria, since mutations in sphingosine-1-phosphate (S1P) lyase are associated with the nephrotic syndrome (74). In Fabry disease, the intracellular pathological accumulation of glycosphingolipids led to cellular and tissue damage, ultimately manifesting in proteinuria and progressive nephropathy. This has been reported with increased urinary B7-1 excretion and podocyturia (75), in the context of native kidney biopsy showing podocyte staining for B7-1. Moreover, the addition of lyso-globotriaosylceramide (lyso-Gb3), present in higher levels in patients with Fabry disease, upregulated the expression of B7-1 mRNA in podocytes *in vitro* (75). The overabundance of sphingolipid was felt to lead *in vivo* to podocyte injury with NF-kappa-B-related B7-1 expression (75). The stimulation of human podocyte TLR3 by poly-IC *in vitro* has also been shown to increase B7-1 mRNA and protein expression in an NF-kappa-B-dependent fashion (72). Thus B7-1 expression appeared to increase with increased levels of NF-kappa-B. Poly-IC injection *in vivo* resulted in proteinuria and increased glomerular expression of B7-1 along with increased urinary B7-1 in mice (76). Podocyte SMPDL3b expression was reduced in biopsies of patients with recurrent FSGS (47). Importantly, SMPDL3b depletion caused a sustained degradation of I-kappa-B-alpha, which led to enhanced NF-kappa-B activity (55). This raised the possibility that the loss of SMPDL3b expression in podocytes in kidneys experiencing recurrence of FSGS (47) may predispose to increased B7-1 expression, possibly NF-kappa-B-mediated. This suggests the intriguing possibility of a connection between sphingolipids and podocyte B7-1 membrane expression in proteinuria. Finally, a recent study using transcriptomic and biological assays in B7-1 transgenic and Adriamycin nephropathy models, found B7-1 mediated podocyte injury through signal transmission to Beta-catenin through a heat shock mediated pathway (Hsp90ab1-LRP-Beta-catenin) (77). This places more biological relevance on the role of B7-1 in podocyte injury. Interestingly, this may also include sphingolipid interaction (76). This area is currently under active study.

### 4.2 Experimental support for SMPDL3b/B7-1 podocyte biomarker model

Several experimental and clinical studies have recently reported similar biomarkers to those in our work. First, in a series of xenotransplant pig-to-primate kidney transplants, with resultant significant proteinuria, both SMPDL3b and B7-1 were expressed on podocytes (78). Rituximab was used for induction with SMPDL3b-demonstrated specificity (53, 78), and B7-1 was also identified on kidney transplant biopsies (78, 79). In their experiments, belatacept was used to treat their primate recipients. While reducing the degree of proteinuria, belatacept did not result in resolution of proteinuria (78). It would be of interest to test whether abatacept may have been more effective in this setting of B7-1 positive podocyte staining in line with our most recent report (70). Secondly, in a rat model of adriamycin-mediated proteinuric kidney disease, studied separately by two different groups: 1) rituximab was found to significantly reduce proteinuria via SMPDL3b-specific modulation (54), and 2) abatacept significantly reduced proteinuria, although B7-1 was not identified on podocytes, and the mechanism was felt to be related to an effect on peripheral blood Th17 lymphocytes (54). Finally, although the molecular mechanisms resulting in nephrotic syndrome are complex (80), podocyte expression of B7-1 has been suggested as a key therapeutic target for Minimal Change Disease (MCD) (71).

Abatacept treatment of a patient with steroid dependent MCD, resulted in sustained remission of proteinuria (81). However, in this study both endothelial cells and podocytes were found to express B7-1 during relapse of MCD (82), again suggesting B7-1 whether expressed on podocytes or EC, could be a therapeutic target. However, a recent report evaluating circulating factors *in vitro* in FSGS plasma demonstrated changes including actin-cytoskeleton rearrangement and excessive formation of reactive oxygen species (ROS) only in podocytes, not in human glomerular microvascular EC (83).

## 5 Approach to rFSGS in B7-1 negative KT recipients

### 5.1 Experience with cGAS-STING in our experimental studies

Although we have observed a reduced incidence of rFSGS in our KT recipients who were treated with a peri-operative dose of rituximab, and subsequently treated with abatacept for those patients identified to express B7-1, we do not yet have effective therapy for those KT recipients who are B7-1 negative. This has led to our next level of investigation, studying the potential role of cGAS-STING in this patient population.

“The cytosol is a highly unusual environment in that it is perhaps the only germ-free location on the planet. In contrast, all extracellular environments have the possibility of being occupied by one or more microbes (84)”.

The protection provided by the innate immune system contributed conceptually to this statement and led to our pursuit of the cGAS-STING axis for potential insight into the B7-1-negative development of rFSGS. Perhaps the putative circulating factors (danger) are being recognized by the cGAS-STING system in a B7-1-independent fashion, ultimately leading to slit diaphragm protein dysfunction and proteinuria. The passage of danger equivalents into the urine may provide a less damaging manner of removing pathogen while minimizing local damage. This suggests that interference with cGAS-STING may lead to new therapeutic possibilities.

STING is an adaptor protein involved in DNA-dependent activation of innate immunity against viral or bacterial infection as well as in autoinflammatory diseases such as vasculopathy and systemic lupus erythematosus (37–39). However, the importance of STING in the regulation of local inflammation in the kidney remains largely unstudied. So, we used this to study mouse models of CKD. This study (37) identifies for the first-time activation of the cGAS-STING signaling pathway in podocytes as a significant contributor to the pathogenesis of glomerular disease of either metabolic (Diabetic kidney disease- DKD) or non-metabolic (Alport Syndrome-AS) origin, suggesting that STING targeting represents a potential therapeutic option to prevent kidney function loss in these conditions. This is particularly important given that there is no available treatment for either DKD (85) or AS (86). Activation of the cGAS-STING pathway has previously been shown in non-immune cells such as mouse embryonic fibroblasts and adipocytes (37). Here, we first confirmed that human as well as murine podocytes express all components of the cGAS-STING signaling pathway both at mRNA and protein levels. This included an increase in gene expression of STING, TBK1 and IRF3, along with increased phosphorylation after treatment with cyclic dinucleotides (c-diAMP).

Terminally differentiated human and murine podocytes were utilized as a model system *in vitro* to investigate expression of the cGAS-STING signaling pathway components and its overall activity. For *in vivo* studies we used db/db type 2 diabetes mice as a model of DKD and Col4a3-/- mice as an experimental Alport syndrome model to determine presence and activity of the cGAS-STING pathway in the kidneys. Activation of STING is associated with proteinuria and podocyte loss in wildtype mice. The cGAS-STING signaling pathway was found to be activated in kidney cortices and glomeruli of db/db mice and mice with AS at baseline and was associated with albuminuria. Pharmacological (with C176) or genetic inhibition ofSTING ameliorated diabetes- or streptozotocin-induced and Alport syndrome-associated glomerular loss of function. Our current findings demonstrate an important role of the cGAS-STING signaling pathway in mediating the detrimental consequences including proteinuria and glomerular dysfunction and point to STING as a potential therapeutic target in glomerular diseases of metabolic and non-metabolic origin (37).

Activation of the cGAS-STING pathway by c-diAMP in podocytes also led to increased expression of type I interferon, a “classic” downstream signal of cGAS-STING. These data confirm the notion that podocytes exhibit features of immune cells and may also be considered as antigen-presenting-like cells. Moreover, increased caspase 3 activity and autophagy markers in c-diAMP treated podocytes suggest that apoptosis and autophagic cell death may be associated with STING activation (37).

While we observed a significant impact of the cGAS-STING pathway on podocyte damage in glomerular diseases of both metabolic (DKD) and non-metabolic (AS and FSGS) origin, the mechanism behind it remains unclear. Some studies indicate that in the kidney, the cGAS-STING pathway may be activated by mitochondrial DNA leakage into the cytosol (40). Thus, it will be important to assess if mitochondrial DNA leakage also occurs in podocytes and contributes to the activation of the cGAS-STING pathway. A recent study suggests that STING is located in the mitochondrial associated membrane, and that this might lead to the specificity of the cellular functions of STING mediated by mitochondrial-ER communication (87). On the other hand, negative regulation of STING signaling is also essential for the prevention of chronic inflammation. Targeting this pathway (cGAS/STING) may represent another therapeutic option to treat or even prevent glomerular disease development and/or progression. We hope that this approach will provide insight into new therapeutic options for those instances of rFSGS that develop despite receiving a peri-operative dose of rituximab, and that are B7-1 negative. Future targets may also include immune markers such as MyD88 (88), CD40 (89), and TNF alpha (90, 91), components of the inflammasome (NLRP1, 3 and 6), RIG-1, MDA5, MAVS, AIM2, RAGE (32, 35), Beta catenin (77) (**Figure 1**), as well as potential markers of adaptive immunity. It is important to note that antibodies to nephrin (92) and CD40 (89) have also been identified as possible culprits in idiopathic nephrotic syndrome and FSGS after kidney transplantation.

## 6 Conclusion

Agents known primarily for their immunosuppressive effects clinically, i.e., calcineurin inhibitors, rituximab and abatacept, may be repurposed, and may impact proteinuria through podocyte-specific effects, rather than or in addition to their immunosuppressive actions (93). This paradigm shift may lead to the development of targeted therapies, guided by tissue-biomarker expression, specifically podocyte expression of SMPDL3-b (94), cGAS-STING (37) and/or B7-1 (95). Circulating factor(s) felt to be involved in the pathogenesis of FSGS may be Danger signal (DAMPs/PAMPs) mimetics (96, 97). We have shown these changes in both specific (membrane biomarkers – SMPDL3b, and B7-1) and non-specific (foot process effacement by electron microscopy) fashion in our series of post-reperfusion kidney transplant biopsies (9, 47, 60, 70). Studying the role of the podocyte in FSGS, specifically the innate immune response, with its expression of TLR’s, inflammasome components, sphingolipids (SMPDL3b) and cGAS-STING and the adaptive immune response, including co-stimulatory molecules (B7-1/CD80, but not B7-2/CD86), may provide important insights into novel immune mechanisms. Hopefully, this will then lead to the discovery of new therapies based on biomarker-driven approaches (98).

It should be noted that the final song (#13, CD3) on Eric Clapton’s live, three-CD set of “Crossroads Revisited” (Eric Clapton and Guests, 2016, Crossroad Concerts LLC, Rhino Entertainment Company) is “Crossroads,” and Robert Johnson is appropriately credited with having written it.

## Author contributions

GB wrote the manuscript. All authors contributed to the article and approved the submitted version.

## References

[1] Sethi S Glasscock RJ Fervenza FC. Focal segmental glomerulosclerosis: towards a better understanding for the practising nephrologist. Nephrol Dial Transplant (2015) 30(3):375–84. doi: 10.1093/ndt/gfu035 24589721

[2] D’Agati VD Kaskel FJ Falk RJ. Medical progress focal segmental glomerulosclerosis. NEngl J Med (2011) 365:2398–411. doi: 10.1056/NEJMra1106556 22187987

[3] Cravedi P Kopp JB Remuzzi G. Recent progress in the pathophysiology and treatment of FSGS recurrence. Amer J Transplant (2013) 13:266–74. doi: 10.1111/ajt.12045 PMC3558619 23312002

[4] DeVriese AS Wetzels JF Glassock RJ Sehti S Fervenza FC. Therapeutic trials in adult FSGS: lessons learned and the road forward. Nat Rev Nephrol (2021) 17:619–30. doi: 10.1038/s41581-021-00427-1 PMC8136112 34017116

[5] Choy BY Chan TM Lai KN. Recurrent glomerulonephritis after kidney transplantation. Am J Transplant (2006) 6:2535–42. doi: 10.1111/j.1600-6143.2006.01502.x 16939521

[6] Hubsch H Montane B Abitbol C Chandar J Shariatmadar S Ciancio G. Recurrent focal l glomerulosclerosis in pediatric renal allografts: the Miami experience. Pediatr Nephrol (2005) 20:210–6. doi: 10.1007/s00467-004-1706-7 15605284

[7] Abbott KC Sawyers ES Oliver JD 3rd Ko CW Kirk AD Welch PG. Graft loss due to recurrent focal segmental glomerulosclerosis in renal transplant recipients in the united states. Am J Kidney Dis (2001) 37:366–73. doi: 10.1053/ajkd.2001.21311 11157379

[8] Garrouste C Canaud G Buchler M Rivalan J Colosio C Martinez F. Rituximab for recurrence of primary focal segmental glomerulosclerosis after kidney transplantation: clinical outcomes. Transplantation (2017) 101(3):649–56. doi: 10.1097/TP.0000000000001160 27043407

[9] Chang JW Pardo V Sageshima J Chen L Tsai HL Reiser J. Podocyte foot process effacement in postreperfusion allograft biopsies correlates with early recurrence of proteinuria in focal segmental glomerulosclerosis. Transplantation (2012) 93(12):1238–44. doi: 10.1097/TP.0b013e318250234a PMC3432300 22499148

[10] Sharma M Sharma R Reddy SR McCarthy ET Savin VJ. Proteinuria after injection of human focal segmental glomerulosclerosis factor. Transplantation (2002) 73(3):366–72. doi: 10.1097/00007890-200202150-00009 11884932

[11] Gallon L Leventhal J Skaro A Kanwar Y Alvarado A. Resolution of recurrent focal segmental glomerulosclerosis after retransplantation. N Engl J Med (2012) 366(17):1648–9. doi: 10.1056/NEJMc1202500 22533598

[12] Kienzl-Wagner K Waldegger S Schneeberger S. Disease recurrence – the sword of Damocles in kidney transplantation for focal segmental glomerulosclerosis. Front Immunol (2019) 10:1669. doi: 10.3389/fimmu.2019.01669 31379860 PMC6652209

[13] Fogo AB. Causes and pathogenesis of focal segmental glomerulosclerosis. Nat Rev Nephrol (2015) 11(2):76–87. doi: 10.1038/nrneph.2014.216 25447132 PMC4772430

[14] Konigshausen E Sellin L. Circulating permeability factors in primary focal segmental glomerulosclerosis: a review of proposed candidates. BioMed Res Int (2016) 9:3765608. doi: 10.1155/2016/3765608 PMC4856884 27200372

[15] Bertelli R Bonanni A Di Donato A Cioni M Ravani P Ghiggeri GM. Regulatory T cells and minimal change nephropathy: in the midst of a complex network. Clin Exp Immunol (2015) 183:166–74. doi: 10.1111/cei.12675 PMC4711159 26147676

[16] Reggiani F Ponticelli C. Focal segmental glomerular sclerosis: do not overlook the role of immune response. J Nephrol (2016) 29:525–34. doi: 10.1007/s40620-016-0272-y 26897173

[17] Zhang YM Gu QH Huang J Qu Z Wang X Meng L-Q. Clinical significance of IgM and C3 glomerular deposition in primary focal glomerulosclerosis. Clin J Am Soc Nephrol (2016) 11(9):1582–9. doi: 10.2215/CJN.01190216 PMC5012474 27340287

[18] LeBerre L Godfrin Y Gunther F Buzelin F Perretto S Smit H. Extrarenal effects on the pathogenesis and relapse of idiopathic nephrotic syndrome in Buffalo/Mna rats. J Clin Invest (2001) 109:491–8. doi: 10.1172/JCI0212858 PMC150869 11854321

[19] De Oliveira JGG Xavier P Carvalho E Ramos JP Magalhaes MC Mendes AA. T Lymphocytes subsets and cytokine production by graft-infiltrating cells in FSGS recurrence post-transplantation. Nephrol Dial Transplant (1999) 14:713–6. doi: 10.1093/ndt/14.3.713 10193825

[20] Benz K Buttner M Dittrich K Campean V Dotsch J Amann K. Characterization of renal immune cell infiltrates in children with nephrotic syndrome. Pediatr Nephrol (2010) 25:1291–8. doi: 10.1007/s00467-010-1507-0 20386928

[21] Pescovitz MD Book BK Sidner RA. Resolution of recurrent focal segmental glomerulosclerosis proteinuria after rituximab treatment. N Eng J Med (2006) 354:1961–3. doi: 10.1056/NEJMc055495 16672715

[22] Dello Strologo L Guzzo I Laurenzi C Vivarelli M Parodi A Barbano G. Use of rituximab in focal glomerulosclerosis relapses after renal transplantation. Transplantation (2009) 88:417–20. doi: 10.1097/TP.0b013e3181aed9d7 19667947

[23] Yabu JM Ho B Scandling JD Vincenti F. Rituximab failed to improve nephrotic syndrome in renal transplant patients with recurrent focal segmental glomerulosclerosis. Am J Transplant (2008) 8:222–7. doi: 10.1111/j.1600-6143.2007.02021.x 17979998

[24] Kufer TA Creagh EM and Bryant CE. Guardians of the cell: effector-triggered immunity steers mammalian immune defense. Trends Immunol (2019) 40(10):939–51. doi: 10.1016/j.it.2019.08.001 31500957

[25] Matzinger P. Tolerance, danger and the extended family. Annu Rev Immunol (1994) 12:991–1045. doi: 10.1146/annurev.iy.12.040194.005015 8011301

[26] Pradeau T Jaeger S Vivier E. The speed of change: towards a discontinuity theory of immunity? Nat Rev Immunol (2013) 13:764–9. doi: 10.1038/nri3521 23995627

[27] Eberl G Pradeau T. Towards a general theory of immunity. Trends Immunol (2018) 39(4):261–3. doi: 10.1016/j.it.2017.11.004 29229264

[28] Chang A KOK Clark MR. The emerging role of the inflammasome in kidney diseases. Curr Opin Nephrol Hypertens (2014) 23:204–10. doi: 10.1097/01.mnh.0000444814.49755.90 PMC4189828 24685591

[29] Yamashita M Millward CA Inoshita H Saikia P Chattopadhyay S Sen GC. Antiviral innate immunity disturbs podocyte cell function. J Innate Immun (2013) 5(3):231–41. doi: 10.1159/000345255 PMC3724426 23296190

[30] Anders HJ Lech M. NOD-like and toll-like receptors or inflammasomes contribute to kidney disease in a canonical and a non-canonical manner. Kidney lnt (2013) 84(2):225–8. doi: 10.1038/ki.2013.122 23903414

[31] Bao W Xia H Liang Y Ye Y lu Y Xu X. Toll-like receptor 9 can be activated by endogenous mitochondrial DNA to induce podocyte apoptosis. Sci Rep (2016) 6:22579. doi: 10.1038/srep22579 26934958 PMC4776276

[32] Xia H Bao W and Shi S. Innate immune activity in glomerular podocytes. Front Immunol (2017) 8:122. doi: 10.3389/fimmu.2017.00122 28228761 PMC5296344

[33] Saurus P Kuusela S Lehtonen E Hyvonen ME Ristola M Fogarty CL. Podocyte apoptosis is prevented by blocking the toll-like receptor pathway. Cell Death Dis (2015) 6:1–12. E1752. doi: 10.1038/cddis.2015.125 PMC4669704 25950482

[34] Zhang C Boini KM Xia M Abais JM Li X Liu Q. Activation of NOD-like receptor protein 3 inflammasomes turns on podocyte injury and glomerulosclerosis in hyper homocysteinemia. Hypertension (2012) 60:154–62. doi: 10.1161/HYPERTENSIONAHA.111 PMC3753400 22647887

[35] Thaiss CA Levy M Itav S Elinav E. Integration of innate immune signaling. Trends Immunol (2016) 37(2):84–101. doi: 10.1016/j.it.2015.12.003 26755064

[36] Loo YM Gale M Jr. Immune signaling by RIG-i-like receptors. Immunity (2011) 34:680–92. doi: 10.1016/j.immuni.2011.05.003 PMC3177755 21616437

[37] Mitofanova A Fontanella AM Tolerico M Mallela S David JM Zuo Y. Activation of stimulator of interferon genes (STING) causes proteinuria and contributes to glomerular diseases. J Am Soc Nephrol (2022) 2021:101286. doi: 10.1681/ASN.2021.101286 PMC9731637 36198430

[38] Guey B Ablasser A. Emerging dimensions of cellular cGAS-STING signaling. Curr Opin Immunol (2022) 74:164–71. doi: 10.1016/j.coi.2022.01.004 35124516

[39] Margolis SR Wilson SC and Vance RE. Evolutionary origins of cGAS-STING signaling. Trends Immunol (2017) 38(10):733–43. doi: 10.1016/j.it.2017.03.004 28416447

[40] Zhong F Liang S and Zhong Z. Emerging role of mitochondrial DNA as a major driver of inflammation and disease progression. Trends Immunol (2019) 40(12):1120–33. doi: 10.1016/j.it.2019.10.008 31744765

[41] Gluck S Ablasser A. Innate immunosensing of DNA in cellular senescence. Curr Opin Immunol (2019) 56:31–6. doi: 10.1016/j.coi.2018.09.013 30296662

[42] Goldwich A Burkard M Olke M Daniel C Amann K Hugo C. Podocytes are nonhematopoietic professional antigen-presenting cells. J Am Soc Nephrol (2014) 24:906–16. doi: 10.1681/ASN.2012020133 PMC3665387 23539760

[43] Reiser J von Gersdorff G Loos M Oh J Asanuma K Giardino L. Induction of B7-1 in podocytes is associated with nephrotic syndrome. J Clin Invest (2004) 113:1390–7. doi: 10.1172/JCI20402 PMC406528 15146236

[44] Moysiadis DK Perysinaki GS Beretsias S Kyriacou K Nakopoulou L Boumpas DT. Early treatment with glucocorticoids or cyclophosphamide retains the slit diaphragm proteins nephrin and podocin in experimental lupus nephritis. Lupus (2012) 21:1196–207. doi: 10.1177/0961203312451784 22767414

[45] Paludan SR. Innate antiviral defenses independent of inducible IFN alpha/beta production. Trends Immunol (2016) 37(9):588–96. doi: 10.1016/j.it.2016.06.003 27345728

[46] Salama AD Pusey CD. Drug insight: rituximab in renal disease and transplantation. Nat Clin Pract Nephrol (2006) 2:221–30. doi: 10.1038/ncpneph0133 16932428

[47] Fornoni A Sageshima J Wei C Merscher-Gomez S Aguillon-Prada R Jauregui AN. Rituximab targets podocytes in recurrent focal segmental glomerulosclerosis. Sci Transl Med (2011) 2011(3):85ra46. doi: 10.1126/scitranslmed.3002231 PMC3719858 21632984

[48] Perosa F Favoino E Caragnano MA Dammacco F. Generation of biologically active linear and cyclic peptides has revealed a unique fine specificity of rituximab and its possible crossreactivity with acid sphingomyelinase-like phosphodiesterase 3b precursor. Blood (2006) 107:1070–7. doi: 10.1182/blood-2005-04-1769 16223774

[49] Bezombes C Grazide S Garret C Fabre C Quillet-Mary A Muller S. Rituximab antiproliferative effect in b-lymphoma cells is associated with acid-sphingomyelinase activation in raft microdomains. Blood (2004) 104:1166–73. doi: 10.1182/blood-2004-01-0277 15126316

[50] Faul C Donnelly M Merscher-Gomez S Chang YH Franz S Delfgaauw J. The actin cystoskeleton of kidney podocytes is a direct target of the antiproteinuric effect of cyclosporine a. Nat Med (2008) 14(9):931–8. doi: 10.1038/nm.1857 PMC4109287 18724379

[51] Chan AC. Rituximab’s new therapeutic target: the podocyte actin cytoskeleton. Sci Trans Med (2011) 3(issue 85):P.85ps21. doi: 10.1126/scitranslmed.3002429 21632983

[52] Yoo TH Pedigo CE Guzman J Correa-Medina M Wei C Villareal R. SMPDL3b expression levels determine podocyte injury phenotypes in glomerular disease. J Am Soc Nephrol (2015) 26(1):133–47. doi: 10.1681/ASN.2013111213 PMC4279736 24925721

[53] Tasaki M Shimizu A Hanekamp I Torabi R Villani V Yamada K. Rituximab treatment prevents the early development of proteinuria following pig-to-baboon xeno-kidney transplantation. J Am Soc Nephrol (2014) 25(4):737–44. doi: 10.1681/ASN.2013040363 PMC3968493 24459229

[54] Takahashi Y Ikezumi Y Saitoh A. Rituximab protects podocytes and exerts anti-proteinuric effects in rat adriamycin-induced nephropathy independent of b-lymphocytes. Nephrology (2017) 22:49–57. doi: 10.1111/nep.12737 26833819

[55] Heinz LX Baumann CL Koberlin MS Snijder B Gawish R Shui G. The lipid-modifying enzyme SMPDL3B negatively regulates innate immunity. Cell Rep (2015) 11:1919–28. doi: 10.1016/j.celrep.2015.05.006 PMC4508342 26095358

[56] Alachkar N Weis C Arend LJ Jackson AM Racusen LC Fornoni A. Podocyte effacement closely links to suPAR levels at time of posttransplantation focal segmental glomerulosclerosis occurrence and improves with therapy. Transplantation (2013) 96(7):649–56. doi: 10.1097/TP.0b013e31829eda4f PMC4026282 23842190

[57] Burke GW Chang J-W Pardo V Sageshima J Chen L Ciancio G. Podocyte foot process effacement in postreperfusion allograft biopsies. Transplantation (2014) 97(7):e38–39. doi: 10.1097/TP.0000000000000053 PMC4745262 24686427

[58] Genovese MC Becker JC Schiff M Luggen M Sherrer Y Kremer J. Abatacept for rheumatoid arthritis refractory to tumor necrosis factor alpha inhibition. N Engl J Med (2005) 353:1114–23. doi: 10.1056/NEJMoa050524 16162882

[59] Wekerle T Grinyo JM. Belatacept: from rational design to clinical application. Transplant Int (2012) 25:139–50. doi: 10.1111/j.1432-2277.2011.01386.x 22151353

[60] Yu CC Fornoni A Weins A Hakroush S Maiguel D Sageshima J. Abatacept in B7-1-positive proteinuric kidney disease. N Engl J Med (2013) 369:2416–23. doi: 10.1056/NEJMoa1304572 PMC3951406 24206430

[61] Baye E Gallazzini M Delville M Legendre C Terzi F Canaud G. The costimulatory receptor B7-1 is not induced in injured podocytes. Kidney Int (2016) 90:1037–44. doi: 10.1016/j.kint.2016.06.022 PMC5073075 27528551

[62] Novelli R Gagliardini E Ruggiero B Benigni A Remuzzi G. Any value of podocyte B7-1 as a biomarker in human MCD and FSGS. Am J Physiol Renal Physiol (2016) 310:F335–41. doi: 10.1152/ajprenal.00510.2015 26697986

[63] Salant DJ. Podocyte expression of B7-1/CD80: is it a reliable biomarker for the treatment of proteinuric kidney diseases with abatacept? J Am Soc Nephrol (2016) 27:963–5. doi: 10.1681/ASN.2015080947 PMC4814196 26400567

[64] Laszik Z Dobi S Vincenti F. B7-1/CD80 is not a reliable immunophenotypical marker of focal segmental glomerulosclerosis, membranous nephropathy, and diabetic nephropathy. J Am Soc Nephrol (2014) 25:TH–OR074.

[65] Larsen CP Messias NC Walker PD. B7-1 immunostaining in proteinuric kidney disease. Am J Kidney Dis (2014) 64(6):999–1005. doi: 10.1053/j.ajkd.2014.07.023 25278092

[66] Grellier J Del Bello A Milongo D Guilbeau-Frugier C Rostaing L Kumar N. Belatacept in recurrent focal segmental glomerulosclerosis after kidney transplantation. Transpl Int (2015) 28:1109–10. doi: 10.1111/tri.12574 25847461

[67] Delville M Baye E Durrbach A Audard V Kofman T Braun L. B7-1 blockade does not improve post-transplant nephrotic syndrome caused by recurrent FSGS. J Am Soc Nephrol (2016) 27(8):2520–7. doi: 10.1681/ASN.2015091002 PMC4978058 26701979

[68] Alachkar N Carter-Monroe N Reiser J. Abatacept in B7-1-positive proteinuric kidney disease. N Engl J Med (2014) 370:1263–4. doi: 10.1056/NEJMc1400502 24670180

[69] Benigni A Gagliardini E Remuzzi G. Abatacept in B7-1-positive proteinuric kidney disease. N Engl J Med (2014) 370:1261–3. doi: 10.1056/NEJMc1400502 24670179

[70] Burke GW III Chandar J Sageshima J Ortigosa-Goggins M Amarapurkar P Mitrofanova A. Benefit of B7-1 staining and abatacept for treatment-resistant post-transplant focal segmental glomerulosclerosis in a predominantly pediatric cohort: time for a reappraisal. Pediatr Nephrol (2022) 38(1):145–159. doi: 10.1007/s00467-022-05549-7 PMC9747833 35507150

[71] Cara-Fuentes G Clapp WL Johnson RJ Garin EH. Pathogenesis of proteinuria in idiopathic minimal change disease: molecular mechanisms. Pediatr Nephrol (2016) 31:2179–89. doi: 10.1007/s00467-016-3379-4 27384691

[72] Shimada M Ishimoto T Lee PY Lanaspa MA Rivard CJ Roncal-Jimenez CA. Toll-like receptor 3 ligands induce CD80 expression in human podocytes via an NF-kappa-B-dependent pathway. Nephrol Dial Transplant (2012) 27:81–9. doi: 10.1093/ndt/gfr271 21617192

[73] Gorelik A LX H Illes K Superi-Furga G Nagar B. Crystal structure of the human alkaline sphingomyelinase provides insights into substrate recognition. J Biol Chem (2017) 292(17):7087–94. doi: 10.1074/jbc.M116.769273 PMC5409475 28292932

[74] Lovric S Goncalves S Gee HY Oskouian B Srinivas H Choi W-I. SGPL1 mutations cause nephrosis with ichthyosis and adrenal insufficiency. J Clin Invest (2017) 127(3):912–28. doi: 10.1172/JCI89626 PMC5330730 28165339

[75] Trimarchi H Canzonieri R Schiel A Costales-Collaguazo C Politei J Stern A. Increased urinary CD80 excretion and podocyturia in fabry disease. J Transl Med (2016) 14:289–96. doi: 10.1186/s12967-016-1049-8 PMC5062834 27733175

[76] Ishimoto T Shimada M Gabriela G Kosugi T Sato W Lee PY. Toll-like receptor 3, polyIC, induces proteinuria and glomerular CD80, and increases urinary CD80 in mice. Nephrol DialTransplant (2013) 28:1439–46. doi: 10.1093/ndt/gfs543 PMC4318967 23262434

[77] Li J Niu J Min W Ai J Lin X Miao J. B7-1 mediates podocyte injury and glomerulosclerosis through communication with Hsp90ab1-LRP-Beta-catenin pathway. Cell Death Differ (2022) 29(12):2399–416. doi: 10.1038/s41418-022001026-8 PMC9750974 35710882

[78] Shah JA Lanaspa MA Tanabe T Watanabe H Johnson RJ Yamada K. Remaining physiological barriers in porcine kidney xenotransplantation: potential pathways behind proteinuria as well as factors related to growth discrepancies following pig-to-kidney xenotransplantation. J Immunol Res (2018), 6413012. doi: 10.1155/2018/6413012 29687010 PMC5857301

[79] Rivard CJ Tanabe T Lanaspa MA Watanabe H Nomura S Andres-Hernando A. Upregulation of CD80 on glomerular podocytes plays an important role in development of proteinuria following pig-to-baboon xeno-renal transplantation – an experimental study. Transpl Int (2018) 31(10):1164–77. doi: 10.1111/tri.13273 PMC6407427 29722117

[80] Purohit S Piani F Ordonez F de Lucas-Collantes C Bauer C Cara-Fuentes G. Molecular mechanisms of proteinuria in minimal change disease. Front Med December (2021) 8:761600. doi: 10.33389/fmed.2021.761600 PMC8733331 35004732

[81] Garin EH Reiser J Cara-Fuentes G Wei C Matar D Wang H. Case series:CTLA4-IgG1 therapy in minimal change disease and focal segmental glomerulosclerosis. Pediatr Nephrol (2015) 30:469–77. doi: 10.1007/s00467-014-2957-6 PMC4869736 25239302

[82] Cara-Fuentes G Venkatareddy M Verma R Segarra A Cleuren AC Martinez-Ramos A. Glomerular endothelial cells and podocytes can express CD80 in patients with minimal change disease during relapse. Pediatr Nephrol 20 June (2020) 35:1887–96. doi: 10.1007/s00467-020-04541-3 PMC8528162 32399663

[83] Veissi ST Smeets B van Wijk JAE Classens R van der Velden TJAM Jeronimus-Klassen A. Circulating permeability factors in focal segmental glomerulosclerosis: in vitro detection. Kidney Int Rep (2022) 7(12):2691–703. doi: 10.1016/j.ekir.2022.09.014 PMC9727530 36506233

[84] Franz KM Kagan JC. Innate immune receptors as competitive determinants of cell fate. Mol Cell (2017) 66:750–60. doi: 10.1016/j.molcel.2017.05.009 PMC5503483 28622520

[85] Luyckx VA Bello AK. Preventing CKD in developed countries. Kidney Int Reports March (2020) 5(3):263–77. doi: 10.1016/j.ekir.2019.12.003 PMC7056854 32154448

[86] Chavez E Rodriguez J Drexler Y Fornoni A. Novel therapies for alport syndrome. Front Med (2022) 9:848389. doi: 10.3389/fmed.2022.848389 PMC9081811 35547199

[87] Xue C Dong N Shan A. Putative role of STING-mitochondria associated membrane crosstalk in immunity. Trends Immunol (2022) 43(7):513–22. doi: 10.1016/j.it.2022.04.011 35637133

[88] Li C Zhang L-M Zhang X Huang X Liu Y Li M-Q. Short-term pharmacological inhibition of MyD88 homodimerization by a novel inhibitor promotes robust allograft tolerance in mouse cardiac and skin transplantation. Transplantation (2017) 101(2):284–93. doi: 10.1097/TP.0000000000001471 27607533

[89] Delville M Sigdel TK Wei C Li L Hsieh S-C Fornoni A. A circulating antibody panel for pre-transplant prediction of FSGS recurrence after kidney transplantation. Sci Trans Med (2014) 6(256):256ra136. doi: 10.1126/scitranslmed.3008538 PMC4394366 25273097

[90] Jain N Khullar B Oswal N Banoth B Joshi P Ravindran B. TLR- mediated albuminuria needs TNF alpha-mediated cooperativity between TLRs present in hematopoietic tissues and CD80 present on non-hematopoietic tissues in mice. Dis Models Mech (2016) 9:707–17. doi: 10.1242/dmm.023440 PMC4920147 27125280

[91] Pedigo CE Ducasa GM Leclercq F Sloan A Mitrofanova A Hashmi T. Local TNF causes NFATc1-dependent cholesterol-mediated podocyte injury. J Clin Invest (2016) 126(9):3336–50. doi: 10.1172/JCI185939 PMC5004940 27482889

[92] Watts AJB Keller KH Lerner G Rosales I Collins AB Sekulic M. Discovery of autoantibodies targeting nephrin in minimal change disease supports a novel autoimmune etiology. J Am Soc Nephrol (2022) 33(1):238–52. doi: 10.1681/ASN.2021060794 PMC8763186 34732507

[93] Salvadori M Tsalouchos A. How immunosuppressive drugs may directly target podocytes in glomerular diseases. Pediatr Nephrol July (2022) 37(7):1431–41. doi: 10.1007/s00467-021-05196-4 34244853

[94] Li G Kidd J Gehr TWB Li P-L. Podocyte sphingolipid signaling in nephrotic syndrome. Cell Physiol Biochem (2021) 55(Suppl 4):13–34. doi: 10.33594/000000356 PMC8193717 33861526

[95] Haraldsson B. A new era of podocyte-targeted therapy for proteinuric kidney disease. N Eng J Med (2013) 369(25):2453–4. doi: 10.1056/NEJMe1312835 24206429

[96] Couser WG Johnson RJ. The etiology of glomerulonephritis: roles of infection and autoimmunity. Kidney Int (2014) 86:905–14. doi: 10.1038/ki.2014.49 24621918

[97] Reiser J Mundel P. Danger signaling by glomerular podocytes defines a novel function of inducible B7-1 in the pathogenesis of nephrotic syndrome. J Am Soc Nephrol (2004) 15(9):2246–8. doi: 10.1097/01.ASN0000136312.46464.33 15339973

[98] Wang C-S Smoyer WE Cara-Fuentes G. Glomerular B7-1 staining: toward precision medicine for treatment of recurrent focal segmental glomerulosclerosis. Pediatr Nephrol (2022) 38(1):13–15. doi: 10.1007/s0047-022-05659-x 35725967

