# Supplementary material for: The podocyte: glomerular sentinel at the crossroads of innate and adaptive immunity
Source: Front Immunol. 2023 Jul 26;14:1201619. doi: 10.3389/fimmu.2023.1201619 (PMC10410139; doi:10.3389/fimmu.2023.1201619)
Supplement: Supplementary file 1 [file DataSheet_1.docx]

SUPPLEMENTAL APPENDIX

Other Rock Musicians who Died at Age 27

1) Brian Jones (Rolling Stones) d. 7/3/1969

2) Alan Wilson (Canned Heat) d. 9/3/1970

3) Jimi Hendrix (The Jimi Hendrix Experience) d. 9/18/1970

4) Janis Joplin (Big Brother and the Holding Company) d. 10/4/1970

1. Jim Morrison (The Doors) d. 7/3/1971
2. Ronald “Pigpen” Charles McKernan (Grateful Dead) d. 3/8/1973
3. Pete Ham (Badfinger) d. 4/24/1975
4. Gary Thain (Uriah Heep) d. 12/8/1975
5. Kurt Cobain (Nirvana) d. 4/5/1994
6. Kristen Pfaff (Hole) d. 6/16/1994
7. Amy Winehouse (Amy Winehouse) d. 7/23/2011
